# Conceptualizing childhood health problems using survey data: a comparison of key indicators

**DOI:** 10.1186/1471-2431-7-40

**Published:** 2007-12-05

**Authors:** Dafna E Kohen, Jamie C Brehaut, Rochelle E Garner, Anton R Miller, Lucyna M Lach, Anne F Klassen, Peter L Rosenbaum

**Affiliations:** 1 Health Information and Research Division, Statistics Canada, Ottawa, Ontario, Canada; 2 Department of Epidemiology and Community Medicine, University of Ottawa, Ottawa, Ontario, Canada; 3 Clinical Epidemiology Unit, Ottawa Health Research Institute, Ottawa, Ontario, Canada; 4 Department of Pediatrics, University of British Columbia, Vancouver, British Columbia, Canada; 5 Centre for Community Child Health Research, Child and Family Research Institute, BC's Children's Hospital, Vancouver, British Columbia, Canada; 6 School of Social Work, McGill University, Montreal, Quebec, Canada; 7 Department of Pediatrics, Faculty of Medicine, McMaster University, Hamilton, Ontario, Canada; 8 CanChild Centre for Childhood Disability Research, McMaster University, Hamilton, Ontario, Canada

## Abstract

**Background:**

Many definitions are being used to conceptualize child health problems. With survey data, commonly used indicators for identifying children with health problems have included chronic condition checklists, measures of activity limitations, elevated service use, and health utility thresholds. This study compares these different indicators in terms of the prevalence rates elicited, and in terms of how the subgroups identified differ.

**Methods:**

Secondary data analyses used data from the National Longitudinal Survey of Children and Youth, which surveyed a nationally representative sample of Canadian children (n = 13,790). Descriptive analyses compared healthy children to those with health problems, as classified by any of the key indicators. Additional analyses examined differences between subgroups of children captured by a single indicator and those described as having health problems by multiple indicators.

**Results:**

This study demonstrates that children captured by any of the indicators had poorer health than healthy children, despite the fact that over half the sample (52.2%) was characterized as having a health problem by at least one indicator. Rates of child ill health differed by indicator; 5.6% had an activity limitation, 9.2% exhibited a severe health difficulty, 31.7% reported a chronic condition, and 36.6% had elevated service use. Further, the four key indicators captured different types of children. Indicator groupings differed on child and socio-demographic factors. Compared to children identified by more than one indicator, those identified only by the severe health difficulty indicator displayed more cognitive problems (p < 0.0001), those identified only by the chronic condition checklist had a greater likelihood of reporting allergies or asthma (p < 0.0001), and those identified as having elevated service use only were more affluent (p = 0.01) and showed better overall health (p < 0.0001). Children identified by only a single indicator were less likely to have serious health problems than those identified by two or more indicators.

**Conclusion:**

We provide information useful to researchers when selecting indicators from survey data to identify children with health problems. Researchers and policy makers need to be aware of the impact of such definitions on prevalence rates as well as on the composition of children classified as being in poor health.

## Background

In Canada, published prevalence estimates of child health problems vary enormously, from 3%–30% for children 0 to 19 years of age [1-6]. In part, this variation is justifiable, because the objectives of survey research vary as do the needs of research users, with emphasis being placed on different components of ill health, including disability [2,7-9], chronic illness [2-5,10-12], well-being [10], or special health care needs [4,13-18]. Another source of variation stems from disagreement regarding how child health problems should be defined, as indicated by the various approaches to categorization (see Additional file 1: Indicators of childhood health problems used in the literature). Yet, while categorizations of child health problems are numerous, most differ primarily in how they incorporate a relatively short list of key health 'indicators'. Using a single large database, the present study examines how these various key indicators characterize children into different health problem groupings.

Scientific and policy-centered discussions of childhood health problems have moved away from a disease-specific (categorical) focus to a more generic (non-categorical) approach [4,12,19-21]. Many of the consequences of childhood health problems are independent of the specific diagnosis since these children and caregivers experience common challenges and life experiences. When a non-categorical approach is used, the consequences of a variety of disorders are explored rather than consequences of any individual disorder [22]. This generic approach has been recommended for use with epidemiological data and has been shown to be valid and reliable [23]. The approach is also useful for program and policy planning where specific diagnoses are not the central focus, and where policy makers could often make better use of comprehensive information that yields rates of child health problems and their consequences (e.g., for the allocation of funds or creation of programs).

Several concepts central to this paper warrant clear definitions. For the present study, we define 'indicators' in a manner consistent with the Canadian Institute for Health Information, that is, "standardized measures by which to compare health status ... and characteristics among different jurisdictions [24]." In contrast, we will use the term 'measures' to refer to health-related variables used in this study as outcomes, to compare groupings by indicator. Finally, the term 'definition' will describe the numerous and varied groupings that can be created by different investigators using different indicators.

In survey research, four key indicators are most commonly used to identify children with health problems. Chronic conditions checklists have been widely used because of the ease with which they can be administered in surveys [1,2], but they are now acknowledged to be insufficient on their own to define health problems because they do not account for health consequences such as disability [21,25,26]. Activity limitation refers to the child's difficulty in performing usual activities; this indicator emphasizes that the functional impact of a chronic condition may or may not be debilitating [5,8,10,27]. Activity limitation is a key component of the International Classification of Functioning [28-30], and it has been widely used in Canadian and international studies of health and disability (see Additional file 1: Indicators of childhood health problems used in the literature). Another approach, used most commonly in the U.S., identifies ill health by the consequences of a condition. For example, Stein [16,21,22,26] focuses on elevated service use (e.g., greater than usual use of health care services, daily use of medication, assistive devices or other technology, medical, psychological, or educational services) as a means of identifying children whose conditions result in important life challenges. Finally, the multi-attribute, utility-based indicator known as the Health Utilities Index (HUI) [31] has also been used to identify health problems. This econometric approach measures a child's ability to function within multiple health domains (attributes) and combines these into a single health utility dimension based on population norms; the utility dimension can then be categorized according to thresholds [32].

Most existing definitions of child health problems differ primarily in the extent to which they make use of these four indicators (see Additional file 1: Indicators of childhood health problems used in the literature). Several studies [10,33,34] consider activity limitations in conjunction with chronic conditions. Others have defined child health problems in terms of activity limitations in conjunction with elevated service use [17,27], while another incorporates chronic conditions and service use [11]. Study of how these definitions differ has been limited. One study compared chronic conditions and activity limitations but did not examine other indicators [5], while another compared four definitions based on one health indicator [1]. To date, no study has compared all four indicators in the context of a single study.

The four key indicators differ dramatically in terms of the number of children that they define as having health problems, yielding definitions that produce substantially different prevalence rates [9,11,16,34,35]. However, because all four have not been examined together systematically, the reasons for these differences remain unclear. One possibility is that the indicators differ primarily in the threshold for which they include children with health problems. For example, chronic condition checklists often capture individuals who have chronic conditions that vary considerably in severity (e.g. asthma), while an activity limitation approach may be a much more conservative approach to categorize child health problems by capturing only those with conditions that impair day-to-day functioning. If the indicators differ primarily in terms of threshold, then the indicators on which a definition is based may be less important than where the threshold is drawn. In contrast, the indicators may differ not only in terms of thresholds, but by systematically including or excluding specific subgroups of children. If so, the most appropriate indicators to use in determining whether a child has a health problem may depend on the outcome of interest and the purpose for which the definition is being used. For example, when deciding how much money to allocate to childhood disability services, one may not want definitions of ill health to include a child with mild asthma, as it might not seem to constitute a disability. In another context where the goal is detecting increases in service use, however, the same child with mild asthma might well appropriately fall into a 'non-healthy' category.

The current study compares four key indicators of childhood health problems using data from a large scale, nationally representative Canadian child survey [36]. If the four indicators differ only in the extent of inclusion through thresholds of full- or ill health, prevalence rates may vary but there should be few systematic differences between indicators in the *kinds *of children categorized as having health problems. However, if the indicators systematically include or exclude certain subgroups of children, it would be helpful to know where the differences exist. Findings from this study will help others to refine their definitions of child health based on outcomes of interest.

## Methods

### Survey

The National Longitudinal Survey of Children and Youth (NLSCY), a long-term study of the physical and social development of Canadian children from birth into early adulthood, provided the source of data for the current study. The NLSCY was started in 1994 and is repeated biennially. The survey is conducted jointly by Human  Resources Social Development Canada. The first cohort of children interviewed in the NLSCY was aged 0 to 11 years in 1994/95. The person most knowledgeable (PMK) of the child provided information on the selected child, as well as information about herself/himself and her/his spouse or partner. In 90% of cases, the PMK was the child's biological mother. In 1994/95, 22,831 children were interviewed in the NLSCY. Children were sampled from all areas of the country proportionate to the regional population. The exception was the northern territories (Yukon, Northwest Territories, Nunavut) which were not included in the NLSCY sample.

### Sample

The sample for the current study consisted of children aged 4 and older from cycle 1 of the NLSCY (n = 13,790); this group was chosen because it has the most complete data in this cycle. Our sample was approximately equally distributed between preschoolers (37.8% ages 4–6) and school aged children (37.1% ages 7–9 and 25.1% ages 10–11), 51.2% were male and most (95%) were born in Canada (Table 1). Mean household income was $52,501 (SD = $91,376) in 1994 Canadian dollars, with 84% of children's parents having at least a high school level education. Eighty-four percent of children were from two-parent families.

**Table 1:** Descriptive statistics of the sample

				Health Problem vs. Healthy Comparison	
				
	Total Sample (n = 13,790)	Health Problem (n = 7196)	Healthy (n = 6594)	t or chi-square tests and p-values	SI
**Child-specific measures**					
Age group					
4–6 years old, %	37.78	37.16	38.45	X^2^_2 _= 1.01, ns	0.03
7–9 years old, %	37.12	37.56	36.63	X^2^_2 _= 0.57, ns	0.02
10–11 years old, %	25.10	25.28	24.92	X^2^_2 _= 0.10, ns	0.01
% male	51.15	54.90	47.06	X^2^_1 _= 34.45, p < 0.0001	0.16
% born in Canada	94.65	95.99	93.19	X^2^_1 _= 12.73, p = 0.0004	0.13
					
**Family-specific measures**					
Income, mean (sd)	$52,501 ($91,376)	$52,544 ($85,800)	$52,454 ($75,658)	t = 0.08, ns	0.00
% PMKs with at least high school education	83.78	83.32	84.28	X^2^_1 _= 0.80, ns	0.03
% Two-parent families	83.88	81.75	86.20	X^2^_1 _= 16.96, p < 0.0001	0.12
					
**Child health status measures**					
% Good/fair/poor health	12.40	19.01	5.19	X^2^_1 _= 277.54, p < 0.0001	0.42
% Injured in past year	11.18	13.92	8.18	X^2^_1 _= 54.68, p < 0.0001	0.18
% Hospitalized in past year	3.81	7.29	0.00	X^2^_1 _= 260.60, p < 0.0001	0.38
% Behaviour problems	29.28	34.37	23.71	X^2^_1 _= 72.79, p < 0.0001	0.23
% Taking medication regularly	10.85	20.79	0.00	X^2^_1 _= 775.29, p < 0.0001	0.67

Presence of a health problem is determined by meeting the criteria of at least one of the four indicators.

### Measures

#### Indicators used in the NLSCY to identify child health problems

(i) For children of all ages, the PMK was asked to report the presence of specific *chronic conditions *that had been diagnosed by a health professional and had lasted, or were expected to last, six-months or more. Specific conditions considered included: asthma, allergies, bronchitis, heart condition or disease, epilepsy, cerebral palsy, kidney condition or disease, mental handicap, or "other". For children aged 6 and older, PMKs also reported whether the child had been diagnosed with a learning disability or any emotional/psychological or nervous difficulties.

(ii)*Activity limitation *was assessed through two questions. In the first case, PMKs reported whether or not the child had any long-term conditions or health problems which prevented or limited their child's participation at school, at play, or in any other activity common for a child of their age. The second question elicited information regarding activity limitations due to the presence of asthma. Children who were reported to be limited by either the general or the asthma-specific limitation question were considered to exhibit activity limitations.

(iii) *Elevated Service Use *was assessed using the Children with Special Health Care Needs (CSHCN) screener which uses a non-categorical approach to identify children with special health care needs based on health and related service use [14]. The five criteria used by the CSHCN screener are: (1) current need or use of prescribed medicine, other than vitamins; (2) need or use of more medical care, mental health, or educational services than is usual for children of the same age; (3) need or receipt of special therapy, such as physical, occupational, or speech therapy; (4) presence of an emotional, developmental, or behavioural problem for which the child needs treatment or counselling; and (5) limitation in the ability to do things most children of the same age can do. To keep this indicator distinct from those previously defined, we did not implement the fifth criterion, i.e. activity limitation, and we did not require any criterion to be specifically tied to a reported long-term condition.

Following Davidoff's (2004) application of the CSHCN criteria to U.S. survey data (National Health Information Survey – Disability), NLSCY items were matched to the concepts underscored by four of the five criteria of the CSHCN Screener (see Additional file 2: CSHCN screener criteria used to define elevated service use). Thresholds for the number of visits in the past year to various health professionals were selected to capture approximately 10 percent or fewer children [37]. The thresholds selected were as follows: six or more visits to a family physician (9.0%), two or more visits to a pediatrician (9.0%), two or more visits to another doctor, such as an orthopedist or an eye specialist (6.5%), one or more visits to a psychiatrist or psychologist (3.6%), or one or more visits to a therapist or counselor (7.5%). Children who met at least one of the criteria were included in the *elevated service *use group.

(iv) The Health Utilities Index (HUI) is a multi-attribute system that classifies an individual's functional ability in eight domains (attributes) of health: vision, hearing, speech, ambulation, dexterity, cognition, emotion and pain. Each attribute is scored from 1 (representing full ability) to a maximum of 5 or 6 (representing poor functional ability). Using an existing algorithm based on population norms, the scores for the eight health domains were combined to produce a composite score that assigned a numeric utility value to the individual's total health state, where 1.0 represented perfect health and 0.0 represented a health state equal to death. Negative utility values are possible on the HUI, representing health states that are considered worse than death [31,38]. The HUI was reported by parents for children aged 4 and older in the NLSCY. Based on cut-off scores developed and validated by others [32], values on the HUI were used to classify children into three groups: those considered healthy, those with minimal health difficulties, and those with *severe health difficulties *(SHD). Children with utility scores 0.97 or greater were considered healthy: a characteristic child receiving this score would be one who is nearsighted and wears glasses, but is otherwise healthy. Those with utility scores lower than 0.81 were considered to have a SHD: for example, a child requiring mechanical support to walk but who does not have pain or other health impediments. Children with scores between 0.97 and 0.81 were considered to have minimal health difficulties. For example, a child who needed glasses to see but was able to walk around the neighbourhood unassisted but with some difficulty received a score of 0.86.

#### Child and Family Characteristics

The PMK was the primary survey respondent and provided information on the following: the child's age (4–6 year olds, 7–9 year-olds, 10–11 year-olds), child's sex, whether the child was born in Canada (yes, no), family income (CDN $), highest education level achieved by the PMK (< high school, ≥ high school), and two parent family structure (yes, no).

#### Child Health Status Measures

PMKs reported on the child's general health on a 5 point scale, where 1 represented "excellent" and 5 "poor" general health. Due to skewed distribution, this scale was dichotomized into "excellent/very good" health vs. "good/fair/poor" health. PMKs also reported whether the child had sustained a serious injury in the past year (yes, no) and whether the child was hospitalized overnight in the past year (yes, no). An additional item indicating whether the child used prescription medications other than vitamins on a regular basis (yes, no) was also included as a measure of child health problems.

The NLSCY includes PMK reports of various behaviour problems for children aged 4 through 11. Responses to 37 questions based on the Child Behavior Checklist (CBCL) but modified for Canadian children and used in other Canadian epidemiological studies [39,40] were used to define the presence of five behaviour scales: hyperactivity/inattention, prosocial behaviour (reversed coded), emotional disorder/anxiety, physical aggression/conduct disorder, and indirect aggression. Based on criteria used elsewhere [41], children who scored 1.5 standard deviations above the mean on any of the five scales were considered to show symptoms of behaviour problems.

### Analyses

Analyses for this study consisted of five components:

1) Descriptive statistics were used to report the child and family characteristics, as well as the prevalence of child health problems in the entire sample.

2) Using a Venn diagram, we depicted the degree to which the four indicators overlapped in their identification of children with health problems. If the four indicators identify entirely unique constructs, we would expect none of the circles in the diagram to overlap. By contrast, if the four indicators measured the same construct but differed only in thresholds, then the indicators would overlap in a series of concentric circles, with the most conservative indicator being captured fully by the next most conservative indicator, and so on.

3) The sample was then subdivided based on whether the children were identified as having a health problem by any of the four key indicators. The distributions of the child and family characteristics and the child health-related measures were compared between the healthy and health problem groups. We hypothesize that if the indicators are valid measures of health problems, those identified by any of the four indicators should score more poorly on the child health measures than healthy children.

4) We used chi-square and t-tests where appropriate to compare children in each of the four indicator groups in terms of available child and family characteristics, and child health measures. Analyses were adjusted for repeated measures, as an individual child may have been identified by more than one indicator, and Bonferroni adjustments were made for all pair-wise comparisons to account for multiple tests. We hypothesized that if the four indicators differed only in terms of the threshold of health problems that they identified, and did not differ in terms of the subgroups of children that they included or excluded, then the four groups should not differ on numerous child or family characteristics, and the child health measures should parallel the group sizes (i.e., those in the smallest group should have the poorest health on average, and those in the largest group should have the best health on average). Deviations from this pattern would suggest that the four key indicators are capturing different types of children.

5) Finally, we created "single indicator only" subgroups that included individuals captured solely by a single indicator (e.g., had a chronic condition, but no limitation, SHD, or elevated service use). We compared the characteristics of those in the single indicator subgroups to individuals who were identified as having a health problem by more than one indicator (i.e., two or more indicators group).

For all estimates, the coefficient of variation (CV) was calculated to determine the quality of the estimate. The coefficient of variation is calculated as the standard error of the estimate divided by the estimate. A CV that is between 0 and 16.5% is considered acceptable. A CV that is between 16.6% and 33.3% is of marginal quality and may have a high level of error. A CV that is greater than 33.3% is considered unacceptable, and such estimates were suppressed in this document and denoted by U in all tables.

For each characteristic, pair-wise comparisons were adjusted by the Bonferroni method to account for the multiple tests. The effect size of comparisons was measured using the standardized increment (SI), which measures the absolute difference between estimates (e.g. means or proportions) over a common standard deviation. Using Cohen's convention of magnitude [42], an SI of 0.2 is considered small, an SI of 0.5 is considered moderate, and an SI of 0.8 is considered large.

Knowledge about how children differ based on classification by single or multiple indicators is informative for researchers and others needing to define such groups. Moreover, it provides a description of children and how they differ based on meeting the criteria for one vs. multiple indicators. All analyses were bootstrapped, which is a method of variance estimation that takes into account the complex sampling design of the survey [43,44].

## Results

Table 1 reports overall sample descriptive statistics, and also subdivides the sample into those children classified as having a health problem by at least one of the four indicators (n = 7196; 52.2% of the entire sample) and those who were classified as not having a health problem by any indicator, i.e., healthy children (n = 6594; 47.8% of entire sample). Compared to those classified as healthy, children with health problems were significantly more likely to be male, more likely to have been born in Canada, and more likely to come from single-parent families. Nevertheless, the effect sizes of these differences are relatively small (0.16, 0.13, and 0.12 respectively). However, in examining the differences in health status measures, children classified as having health problems had significantly poorer ratings on all health status measures, and the effect sizes of these differences were small to moderate.

**Figure 1:**
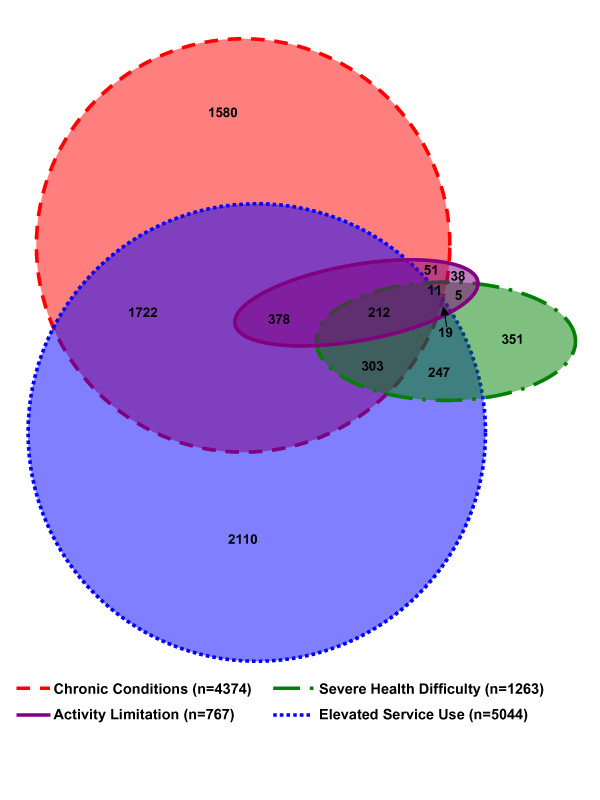
**Venn diagram of overlap between four indicators of ill health, n = 7196**. The circles denote each of the four indicators and are proportional in size to the sample captured by each; the intersections are not proportional. Colour legend: Red = Chronic Conditions, Purple = Activity Limitations, Green = Severe Health Difficulty, Blue = Elevated Service Use. Not shown on the diagram is the intersection between activity limitations and elevated service use (n = 53) and the intersection between chronic conditions and severe health difficulties (n = 116).

In Figure 1, a Venn diagram was used to depict graphically children with health problems in terms of the proportion identified by each of the four key indicators, as well as the proportion identified by more than one indicator. The area encompassed by all four circles represents all 7196 children defined as having health problems. The area of each circle corresponds to the number of individuals identified as having health problems by that particular indicator, while the overlap between circles approximately describes the proportion of individuals identified by more than one indicator. The diagram shows both considerable overlap between certain indicators and comparatively little overlap between others. Of the 767 individuals identified as having an activity limitation, only 5.0% (38/767) were not captured by any other indicator, signifying that the vast majority of those with activity limitations are also found to have a health problem by at least one other indicator. By contrast, the other three indicators identified proportionately much larger 'single indicator only' groups: 27.8% (351/1263) of the SHD group was captured only by that construct, 36.1% (1580/4374) for chronic conditions, and 41.8% (2110/5044) for elevated service use. Overall, over half the children in our sample of children with health problems (56.7% or 4080/7196) were captured by only one of the four indicators, while fewer (43.3% or 3116/7196) were captured by two or more indicators.

Table 2 describes and compares the characteristics of children identified by each of the four indicators. One child characteristic (i.e. age) and three family characteristics (i.e. income, parental education, and number of parents) distinguished the children between groups. Children identified by the elevated service use indicator were more likely to be younger than children in the other groups (40.5% 4-6 years versus 23%–33% for the other indicator groups, SIs ranging from 0.15 to 0.36), while children with SHDs were significantly older than the others (35.9% 10-11 year olds versus 23%–28% for the other indicator groups, SIs ranging from 0.18 to 0.30). Children with SHDs were also from families with significantly lower household incomes (mean income = $44,498, sd = $42,423, SIs ranging from 0.10 to 0.13), were more frequently from families with caregivers with less than a high school education (22.41% versus 19.71%, 16.46% and 16.01%, for the other indicator groups, SIs ranging from 0.16 to 0.17), and were less frequently from two-parent families than the other groups (73.63% versus 76.67%, 79.79% and 82.26% for the other indicator groups, SIs ranging from 0.15 to 0.22). Lastly, children with chronic conditions were significantly less likely than others to be from single-parent families (26.37% versus 23.33%, 20.21% and 17.74%, for the other indicator groups, SIs ranging from 0.06 to 0.22).

**Table 2:** Descriptive statistics for individuals captured by each indicator and comparison across indicator categories (n = 7196)

	A: Activity Limitations (n = 767)		B: Severe Health Difficulty (n = 1263)		C: Chronic Conditions (n = 4374)		D: Elevated Service Use (n = 5044)		
		
	Estimate	SI	Estimate	SI	Estimate	SI	Estimate	SI	Overall Comparison
**Child-specific measures**									
Age group									
4–6 years old, %	32.29^BD^	^B^0.20	23.34^ACD^	^A^0.20	33.27^BD^	^B^0.21	40.50^ABC^	^A^0.17	X^2^_3 _= 117.40, p < 0.0001
		^D^0.17		^C^0.21		^D^0.15		^B^0.36	
				^D^0.36				^C^0.15	
7–9 years old, %	40.63		40.81		38.98		36.78		X^2^_3 _= 8.80, p = 0.03
10–11 years old, %	27.08^BD^	^B^0.19	35.85^AC^	^A^0.19	27.74^BD^	^B^0.18	22.72^ABC^	^A^0.10	X^2^_3 _= 57.62, p < 0.0001
		^D^0.10		^C^0.18		^D^0.12		^B^0.30	
				^D^0.30				^C^0.12	
% male	60.46		58.54		57.43		55.43		X^2^_3 _= 6.29, ns
% born in Canada	95.72		94.38		96.64		96.21		X^2^_3 _= 2.95, ns
**Family-specific measures**									
Income, mean (sd)	51,845^B ^(72,089)	^B^0.13	44,498^ACD ^(42,423)	^A^0.13	51,748^B ^(63,859)	^B^0.12	53,009^B ^(90,639)	^B^0.10	F_3,4262 _= 11.08 p < 0.0001
				^C^0.12					
				^D^0.10					
% PMKs with at least high school education	80.29		77.59^CD^	^C^0.16	83.54^B^	^B^0.16	83.99^B^	^B^0.17	X^2^_3 _= 13.68 p = 0.004
				^D^0.17					
% Two-parent families	76.67		73.63^CD^	^C^0.22	82.26^BD^	^B^0.22	79.79^BC^	^B^0.15	X^2^_3 _= 24.70 p < 0.0001
				^D^0.15		^D^0.06		^C^0.06	
**Child health status measures**									
% Excellent/very good health	53.62^BCD^	^B^0.38	71.79^AD^	^A^0.38	76.44^A^	^A^0.51	77.95^AB^	^A^0.56	X^2^_3 _= 88.51 p < 0.0001
		^C^0.51		^D^0.15				^B^0.15	
		^D^0.56							
% Injured in past year	15.29		14.48		13.65		15.40		X^2^_3 _= 5.72, ns
% Hospitalized in past year	15.74^BCD^	^B^0.23	8.46^A^	^A^0.23	7.14^AD^	^A^0.31	10.40^AC^	^A^0.17	X^2^_3 _= 104.41 p < 0.0001
		^C^0.31				^D^0.11		^C^0.11	
		^D^0.17							
% Behaviour problems	41.76^BCD^	^B^0.41	62.12^ACD^	^A^0.41	34.94^AB^	^A^0.14	35.01^AB^	^A^0.14	X^2^_3 _= 144.11 p < 0.0001
		^C^0.14		^C^0.55		^B^0.55		^B^0.55	
		^D^0.14		^D^0.55					
% Taking medication regularly	58.28^BCD^	^B^0.77	21.38^ACD^	^A^0.77	29.82^AB^	^A^0.60	29.66^AB^	^A^0.61	X^2^_3 _= 144.02 p < 0.0001
		^C^0.60		^C^0.19		^B^0.19		^B^0.18	
		^D^0.61		^D^0.18					

Children may be identified by more than one indicator category. Superscript letters indicate significant differences (adjusted for multiple comparisons, p < 0.01) between the value and the corresponding value from the column denoted by the letter. Effect sizes (standardized increments) are given only for those comparisons that were found to be statistically significant.

Child health measures differed between groups. PMKs' ratings of the child's general health mirrored group size, with the smallest group (Activity Limitation; n = 767) having the lowest proportion of children with excellent/very good health (53.6%), and the largest group (Elevated Service Use; n = 5044) having the highest proportion (78.0%). Other health status characteristics, however, did not conform to this pattern. The proportion of individuals with a serious injury in the past year did not vary between groups. The proportion hospitalized in the past year was highest for those in the Activity Limitation group (15.7%), while those in the SHD and Chronic Conditions groups showed the lowest rates (8.5% and 7.1% respectively; these rates did not differ significantly). The proportion of reported behaviour problems was highest among the SHD group (62.1%) and lowest for the Chronic Conditions and Elevated Service Use groups (34.9% and 35.0%, respectively). The proportion of children taking medications regularly was highest for the Activity Limitations group (58.3%), and lowest for the SHD group (21.4%). These findings suggest that children in the Activity Limitation group had the poorest health while children in the Elevated Service Use group did not necessarily experience large health implications, even though they accessed the health care system and health resources more often than other children.

Table 3 compares the characteristics of children in the "single indicator" subgroups to those in the "two or more indicators" group. The Activity Limitation Only subgroup was very small (n = 38), resulting in low power to detect statistically significant differences and suppression of some estimates.

**Table 3:** Characteristics of those identified by a single indicator only compared with those identified by two or more indicators (n = 7196)

	Activity limitation only (n = 38)		Severe health difficulty only (n = 351)		Chronic condition only (n = 1580)		Elevated service use only (n = 2110)		Two or more indicators (n = 3117)
		
	Estimate	SI^†^	Estimate	SI	Estimate	SI	Estimate	SI	
**Child-specific measures**									
Age group									
4–6 years old, %	U	U	25.62	0.18	31.65	0.05	48.03*	0.29	34.05
7–9 years old, %	62.05	0.48	38.80	0.00	38.64	0.00	34.55	0.08	38.61
10–11 years old, %	U	U	35.58	0.18	29.71	0.05	17.42*	0.23	27.34
% male	46.59	0.27	57.55	0.05	53.47*	0.13	48.34*	0.23	59.88
% born in Canada	87.09	0.51	88.20	0.41	96.79	0.01	96.05	0.03	96.54
**Family-specific measures**									
Income, mean (sd)	$43,337 ($51,761)	0.11	$45,429 ($47,724)	0.08	$53,780 ($58,920)	0.06	$56,239* ($103,209)	0.07	$50,330 ($61,640)
% PMKs with at least high school education	60.17	0.58	74.42	0.21	84.34	0.05	85.68	0.09	82.49
% Two-parent families	87.08	0.21	84.87	0.15	87.29*	0.23	81.77	0.08	78.52
**Child health status measures**									
% Good/fair/poor health	U	U	18.58*	0.25	9.21*	0.49	10.24*	0.48	30.02
% Injured in past year	U	U	7.73*	0.22	9.71*	0.17	15.83	0.01	15.56
% Hospitalized in past year	0.00	...	0.00	...	0.00	...	8.48	0.09	11.10
% Behaviour problems	U		52.83	0.22	25.98*	0.33	26.69*	0.32	41.97
% Taking medication regularly	0.00		0.00	...	0.00	...	7.06*	0.80	43.21
**Other measures**									
% reporting asthma or allergies	0.00	...	0.00	...	81.83*	0.32	0.00	...	67.38
% reporting "other" condition	0.00	...	0.00	...	10.70*	0.13	0.00	...	15.30
% with level 4 cognitive deficit (HUI)	0.00	...	72.08*	1.35	0.00	...	0.00	...	16.29
% with mild health difficulty (HUI cut-point)	U	U	0.00	...	23.36	0.01	26.07	0.08	22.73
Num. doctors visits in past year, mean (sd)	1.59* (1.77)	0.40	1.47* (2.20)	0.42	2.01* (2.23)	0.46	6.84* (12.89)	0.13	9.11 (19.08)
Num. doctors consulted in past year, mean (sd)	1.08* (0.70)	0.45	0.89* (1.24)	0.57	1.11* (1.22)	0.47	1.70* (1.41)	0.09	1.84 (1.71)

All comparisons made pair-wise with those classified by two or more indicators. By definition, individuals in the uniquely defined indicator groups often did not possess a particular characteristic; in such cases, significance of the difference with the "two or more indicators" group was not assessed. Statistical significance was adjusted for multiple comparisons (significant differences, *p < 0.0125).

^†^Effects sizes (standardized increments) refer to comparisons made with "two or more indicators" group

... Effect sizes (standardized increments) not calculated when proportion was zero in one of the two groups.

These estimates should be interpreted with caution due to marginal coefficient of variation (CV, 16.6–33.3%). Estimates whose CV is greater than 33.3% are unacceptable for publication and have been suppressed (U)

Children in the SHD Only subgroup (n = 351) did not differ from the Two or More Indicators group on any of the child- or family-specific measures. However, they were significantly more likely to be rated by their PMKs as being in excellent or very good health (81.4% vs. 70.0%, SI = 0.25) and less likely to have been injured in the past year (7.7% vs. 15.6%, SI = 0.22) than those identified by two or more indicators, with those identified by two  or more indicators, with small effect sizes. Those in the SHD Only subgroup were also significantly more likely to show symptoms of a cognitive deficit as measured by the HUI than those in the Two or More Indicators group (72.1% vs. 16.3%, SI = 1.35).

Children in the Chronic Conditions Only subgroup (n = 1580) were more frequently from two-parent families than children in the Two or More Indicators subgroup (87.3% vs. 78.5%, SI = 0.23) and showed better health (excellent/very good health 90.8% vs. 70.0%, SI = 0.49) and less frequent injury (9.7% vs. 15.6%, SI = 0.17). Among the chronic conditions reported, 81.8% of children in the Chronic Conditions Only subgroup reported being diagnosed with asthma and/or allergies, a significantly higher proportion than among children in the Two or More Indicators subgroup (67.4%, SI = 0.32).

Finally, the Elevated Service Use Only subgroup (n = 2110) consisted of children who were younger, more likely to be female (51.7% vs. 40.1%, SI = 0.23), possessed higher levels of family income ($56,239 vs. $50,330, SI = 0.07), and had better health (excellent/very good health, 89.8% vs. 70.0%, SI = 0.48; fewer behaviour problems, 26.7% vs. 42.0%, SI = 0.32; regular medication, 7.1% vs. 43.2%, SI = 0.80) than those in the Two or More Indicators subgroup. They also had fewer doctor visits and consulted fewer physicians in the past year than those in the Two or More Indicators group (6.8 vs. 9.1 visits, SI = 0.13; 1.7 vs. 1.8 physicians, SI = 0.09, respectively).

## Discussion

The task of identifying childhood health problems is one that many have struggled with, often without detailed evidence on which to base decisions. Current definitions tend to incorporate some combination of four key indicators. This study shows that these four key indicators differ considerably in terms of the children that they classify as having health problems. Of the 7196 individuals identified in our sample as having a health problem, over half (56.7%) were identified only by a single indicator and were classified as healthy by the other three indicators. These 'single indicator' children differ from 'two or more indicator' children (i.e., those individuals for whom there is some agreement on multiple indicators of the presence of a health problem) both demographically and in terms of health status measures. The choice of whether to consider these single indicator children as having a health problem is not obvious: excluding them will dramatically reduce prevalence rates as well as sample size, while including them may change the composition of the group by including children with varying levels of severity. We examined these different groups in order to provide some evidence upon which to base decisions about categorizing children with health problems.

By considering every child that is identified as having a health problem by any of the four key indicators, one is left with an extremely liberal definition of ill health, one that categorizes over half (52.2%) of the population as having health problems. One might argue that such a definition is so broad that it becomes meaningless, yet Table 1 shows that even this very broad grouping shows substantially poorer health on a variety of outcomes as compared to those not identified by any indicator, i.e. healthy children. In contrast, differences in child and family demographic characteristics were relatively small, with the exception of a bias towards a higher proportion of males in the health problem group. This last result is consistent with previous findings that, in general, males have more health problems than females [45,46]. Choosing this method to define health problems may therefore be a defensible approach, particularly in situations where sample sizes are small and a categorization method that maximizes the numbers of people in the health problems group is required.

Many analyses of child health attempt to develop their definitions of childhood health problems or disability by combining one or more key indicators with an 'AND' rule (e.g. to be considered disabled, the child must have a chronic condition AND an activity limitation [10]). In part, this practice stems from the assumption that those who are categorized by only one indicator may be less likely to be truly impaired than those who are captured by several indicators, yet this assumption has never been tested.

Our findings show two key points about such 'AND' rule definitions. First, because of the considerable non-overlap of the four indicators, 'AND' definitions dramatically reduce the target sample size. For example, examination of Figure 1 shows that the combination of chronic conditions AND high service use yields a sample size of 2615 (1722+303+212+378), which is only 38% of the 6803 children yielded by a chronic conditions OR elevated service use definition (4374+5044-2615 = 6803 unique children). Second, our findings show that subgroups that are excluded from 'AND' rules (e.g. the first four columns in Table 3) score lower (i.e., more poorly) on a variety of health measures than do healthy children (Table 1). As such, excluding 'single indicator' individuals from definitions of health problems and including them along with 'healthy' individuals will result in an underestimation of the health differences between the two groups. Without careful consideration then, use of an 'AND' rule can result in a substantially reduced sample size, and an underestimate of the health effects between groups. Incorporation of multiple groups (i.e., healthy, single indicator, two or more indicators) should be considered.

For many purposes, adoption of the most liberal criterion of ill health (i.e. any one or more of the four key indicators) may be inappropriate. If so, other criteria based on these key indicators may be conceptualized as systematically including or excluding different parts of the Venn diagram presented in Figure 1. By describing these subgroups, we have provided information that is useful in understanding whether to include or exclude such subgroups from definitions of health problems. In a four-circle Venn diagram, there are up to 16 mutually exclusive areas of overlap. While describing how all 16 subgroups differ in our sample may be interesting and informative, it is beyond the scope of the current paper due to complexity and small sample sizes for certain subgroups. Instead, we chose to focus on five groups of particular interest: the four 'single indicator' groups (because any 'AND' rule will automatically exclude these groups), and the combination of all the groups that overlap with two or more indicators, collectively referred to as the 'two or more indicators' group. In the following section we summarize what our findings suggest will be the effects of subgroup inclusion on the size and composition of such groups.

### Children identified as having only an Activity Limitation

The Activity Limitation Only group comprised a tiny fraction of our overall sample (38/7196, or 0.5%). This is sensible, as over half (52.8%) reported behavior  problems. The reasons why these 38 individuals were not captured by any other construct (i.e., how these 38 differ from the others) cannot be determined by available data; as Table 3 shows, this group is relatively unremarkable on all of the measures considered in the present study. One might have expected an association of activity limitations with acute conditions such as injuries, yet the rate of injuries and hospitalizations had to be suppressed for this group, as they were too low to report. We hypothesize that this group may include some children misreported as having a limitation, or who may have a current condition giving rise to a limitation that has not yet been diagnosed, although this is an area for further inquiry. Because the number of 'activity limitation only' children is low, and because this group was not distinctive on the measures we examined, including or excluding individuals who report activity limitations only will not greatly affect the size or composition of a subgroup of children with health problems. This assumes, however, that other datasets have similar proportions of these kinds of individuals.

### Children identified as having only a Severe Health Difficulty

The Severe Health Difficulty Only group in our sample comprised 4.9% of the entire sample (351/7196). While this group is not a large component of the total possible sample and does not differ demographically from the Two or More Indicators subgroup, these 351 children differed from those in the Two or More Indicators subgroup in that they are more frequently reported as having psychological as opposed to physical problems; over half reported behaviour problems, while 72% reported a level four cognitive deficit on the HUI (on a 6 point scale, defined as "somewhat forgetful and has a little difficulty when trying to think or solve day to day problems"), both of which were substantially higher than any other of the subgroups in Table 3.

It should be noted that the HUI, like most survey overview instruments, may have limited ability to incorporate detailed perspectives on the cognitive deficits or behavioural problems associated with children and youth whose status is judged to be impaired in some way. This may affect the way the overall health utilities are judged. Even so, none of the other indicators as implemented by the NLSCY specifically consider cognitive deficits. Researchers should therefore decide whether the target sample should include children with cognitive deficits and behaviour problems. If so, approaches such as the HUI reported here, or specific items targeted at assessing these areas, should be incorporated into the definition of health problems.

### Children identified as having only a Chronic Condition (checklist approach)

The Chronic Condition Only subgroup comprised a much larger proportion of the total sample (1580/7196 or 22.0%). As such, inclusion or exclusion of this subgroup has the potential to affect the overall size of the target sample dramatically. There were some indications of a demographic bias, although effects were modest. Compared to the Two or More Indicators subgroup, the Chronic Conditions Only subgroup was more likely to come from two-parent families, and included fewer males. Perhaps more important is the substantially greater likelihood of reporting allergies or asthma in the Chronic Conditions Only subgroup. This is consistent with previous findings showing that chronic condition checklists produce high prevalence estimates compared to other definitions of health problems [16], in part because asthma and allergies are both very common and, in many cases, relatively mild in terms of their impact on daily functioning (and thus are less likely to be captured by the other indicators). This hypothesis is supported in our data by the fact that the Chronic Conditions Only subgroup showed the best overall health measures of all subgroups (Table 3). When considering whether to include this subgroup in the target sample, one should therefore consider whether a relatively large subgroup that may include a number of relatively mild cases of conditions such as allergies and asthma is appropriate to the purpose for which the data are being used.

### Children identified as having only Elevated Service Use

The Elevated Service Use Only subgroup was also quite large, comprising 29.3% (2110/7196) of the total sample. Children in this group also differed from those in the Two or More Indicators group in terms of demographics: this subgroup was younger, included fewer males and came from families with substantially higher incomes. This sub-sample showed better health ratings, more frequent 'mild health difficulties', lower medication use, and fewer overall doctor visits than the Two or More Indicators group. In short, this very large subgroup is the most affluent and healthiest subgroup that we examined. It is more likely to include children with relatively mild conditions, or conditions that have not been diagnosed because of the age of the child, yet are more likely to be living in affluent families. These findings are consistent with the notion that relatively affluent families have the resources and access to care to make use of services for more mild childhood problems. Inclusion of this subgroup in the target subgroup will result in a large number of children with mild conditions; only the most inclusive of definitions should likely include this subgroup.

### Children identified by two or more indicators

Individuals for whom there was agreement on at least two indicators of a health problem were included in the Two or More Indicators group. Despite how large some of the single indicator groups were, the two or more group was even larger, comprising 43.3% (3117/7196) of the entire health problems sample. More importantly, this group reported the poorest health overall, showing the poorest health ratings, highest rate of hospitalizations and medication use, and the largest number of health professionals visited and the most numerous visits. In short, the current findings support the previously unfounded assumption that individuals are likely to have less serious health problems if they satisfy only one of the four key indicator conditions than if they satisfy more than one. Because of its relatively large size, and poor overall health, this subgroup could serve as a meaningful grouping for the identification of children in poor health in a variety of situations.

### Limitations

Several study limitations warrant consideration. All indicators were based on parent reported measures that may not accurately reflect the child's health problems; physician diagnoses and data linked from administrative sources would be a useful area for further study. Moreover, none of the indicators specifically include behaviour problems, yet this is an important aspect of child health. Chronic conditions lists typically exclude rare conditions and fail to capture other undiagnosed conditions; again, alternate, more detailed sources of information on child health problems could overcome this weakness. Information on activity limitations was based on merely two survey items; detailed instruments measuring functional status and limitations exist and should be considered in future surveys [47-50]. Finally, we broadly categorized children as healthy or as unhealthy as classified by a single indicator or two or more indicators of health problems. A more detailed examination of the extent to which select subgroups differ (e.g. children with chronic conditions with and without activity limitations; children with severe health difficulties and high service use) may also help those attempting to identify groupings of children with health problems using survey items. Such subgroup analyses were beyond the scope of the present study and should be pursued in future work.

## Conclusion

Defining health problems requires both a clearly defined objective for asking the question(s), and an understanding about how different indicators can be combined to produce a target sample of adequate size and appropriate composition. This study examined how the four key indicators most commonly involved in defining poor child health differ in the way they classify children. Of the 7196 children classified as having a health problem by any indicator, less than half were classified that way by more than one indicator. Those for which there was some agreement between indicators showed the poorest overall health. Children who had only a chronic condition or only  elevated service use tended to come from more affluent backgrounds and have milder health problems; those who fit the criterion of SHD came from less affluent backgrounds and exhibited more cognitive deficits and behaviour problems. Even the most liberal definition of health problems, i.e. those identified by any one of the four indicators, was related to poorer child health measures as compared to healthy children. Both our analyses and conclusions warrant replication. Future research could explore multiple definitions of child health problems using different data sources, including international data. For example, the National Health Information Survey and the National Longitudinal Survey of Youth in the United States, the Health Survey for England, and the Scottish Household Survey all address issues of child health and may have additional definitions that could be explored.

Researchers and policy-makers should be aware of the wildly different prevalence rates and sample characteristics that different definitions of health problems provide, and should incorporate this knowledge when considering definitions of childhood health problems for various purposes.

## Abbreviations

HUI: Health Utilities Index; NLSCY: National Longitudinal Survey of Children and Youth; PMK: Person most knowledgeable; CSHCN: Children with special health care needs; SHD: Severe health difficulties; CBCL: Child behaviour checklist; QuICCC-R: Questionnaire for Identifying Children with Chronic Conditions – Revised; ICF: International Classification of Functioning.

## Competing interests

The author(s) declare that they have no competing interests.

## Authors' contributions

DK, JB and RG participated in the design of the study, performed the statistical analysis and took the lead in drafting the manuscript. AM, LL, AK and PR conceived of the study, and participated in its design and coordination and helped to draft the manuscript. All authors read and approved the final manuscript.

## Pre-publication history

The pre-publication history for this paper can be accessed here:



## Supplementary Material

Additional file 1 **Indicators of childhood health problems used in the literature**. Appendix summary of relevant studies. Click here for file

Additional file 2 **Children with Special Health Care Needs (CSHCN) screener criteria used to define elevated service use**. Appendix details of the items used for criteria for elevated service use. Click here for file
