# Supplementary material for: Conceptualizing childhood health problems using survey data: a comparison of key indicators
Source: BMC Pediatr. 2007 Dec 5;7:40. doi: 10.1186/1471-2431-7-40 (PMC2248574; doi:10.1186/1471-2431-7-40)
Supplement: Additional file 1 — Indicators of childhood health problems used in the literature. Appendix summary of relevant studies. [file 1471-2431-7-40-S1.doc]

### Additional File 1: Indicators of childhood health problems used in the literature

| **Reference** | **Indicators** | **Findings / Recommendations** |
| --- | --- | --- |
| [14] | CSHCN Screener | CSHCN Screener captures only children who have current health needs, not those at risk for developing health difficulties in the future. |
| [15] | CSHCN Screener  QuICCC-R | The children identified by the CSHCN screener tend to have greater health limitations that the children identified by the QuICCC-R |
| [10] | Chronic condition (CC), checklist approach plus several HUI domains  Activity limitation, single item | Allergy or hay fever not included in list of chronic conditions; Asthma was included  Selective list of chronic conditions used “represents most of the clinically significant, moderate to severe chronic problems in Canadian children.”  Limitations defined to “measure moderate to severe disability of long duration” |
| [7] | Functional or activity limitations | Authors used “…an operational definition that allows researchers to capture the largest group of children with any sort of limitation while using a limited set of identifying questions.” |
| [37] | CSHCN Screener | Screener does not address the issue of severity of the condition or degree of impact on family |
| [8] | Functional limitations  (4 domains)  Societal limitation | Authors state that strength of their measurement is that it maintains a distinction between functional limitation, disability and societal limitation. |
| [33] | Chronic conditions (checklist)  Activity limitation | Recognition that certain types of activity limitations may not constitute disabilities and vice versa (e.g. learning disabilities not always identified by activity limitation) |
| [11] | Chronic condition (using ICD9 codes)  QuICCC | Those identified only by the QuICCC may not have a specific diagnosis, but have symptoms that increase their use of the health system |
| [50] | Chronic condition  Functional limitation | Various combinations of chronic conditions and functional limitations were used to identify individuals with mental retardation and developmental disabilities; “Mental retardation and developmental disabilities are not interchangeable classifications” (p.248) |
| [34] | Chronic conditions  Activity limitations  ICF concepts | Many surveys do not include chronic conditions that capture neoplasms (i.e. cancer), endocrine/nutritional or metabolic diseases (e.g. diabetes, cystic fibrosis), or diseases of the musculoskeletal system and connective tissue (e.g. muscular dystrophy); Authors recommend to include follow-up questions to assess the duration and severity of impairments; Recommendation to include a follow-up question regarding the cause of the activity limitation, rather than making it part of the question, particularly to capture persons who are limited but not due to a chronic condition |
| [5] | Chronic condition, checklist  Physical impairment (HUI)  Activity limitation | Recommend to keep chronic conditions and physical impairment separate in future research |
| [35] | U.S. Maternal & Child Health Bureau’s definition | Since not all children with elevated health care needs actually receive services, it may be necessary to identify children with presumed need for elevated service use, even if services are not accessed |
| [9] | Activity limitation    Reduction in time spent in activity participation | Authors note that the same questions may produce very different results due to the following: (i) sampling variation; (ii) subjective vs. objective questions; (iii) survey context; (iv) placement of disability questions within survey; (v) use of proxy respondents; (vi) language and culture |
| [16] | Chronic condition  QuICCC | QuICCC excludes children who are not currently experiencing consequences of their health conditions; QuICCC also tends to exclude children with conditions not considered to be chronic (e.g. hay fever) |
| [18] | CSHCN Screener | Among those identified as having special health care need, many reported inability to access necessary services. No reports on unmet needs among those not identified by the Screener  i.e. may need to identify those presumed to have special needs apart from access of services |
