# Supplementary material for: Conceptualizing childhood health problems using survey data: a comparison of key indicators
Source: BMC Pediatr. 2007 Dec 5;7:40. doi: 10.1186/1471-2431-7-40 (PMC2248574; doi:10.1186/1471-2431-7-40)
Supplement: Additional file 2 — Children with Special Health Care Needs (CSHCN) screener criteria used to define elevated service use. Appendix details of the items used for criteria for elevated service use. [file 1471-2431-7-40-S2.doc]

### Additional File 2: CSHCN screener criteria used to define elevated service use

| **Criterion 1: Child currently needs or uses medicine prescribed by a doctor, other than vitamins** |
| --- |
| Use of prescription medication on regular basis |
| **Criterion 2: Child needs or uses more medical care, mental health or educational services than is usual for most children of the same age** |
| Requires mechanical support (e.g. braces, cane, crutches) to be able to walk around the neighbourhood |
| Requires a wheelchair to get around |
| Requires special equipment (e.g. devices to assist in dressing) because of limitations in the use of hands or fingers? |
| Requires use of a hearing aid to hear |
| Six or more visits to a family doctor, past 12 months |
| Two or more visits to a pediatrician, past 12 months |
| Two or more visits to another medical doctor, past 12 months |
| One or more overnight stays in hospital, past 12 months |
| Receives special education because a physical, emotional, behavioural, or some other problem limits the kind or amount of schoolwork he/she can do? |
| **Criterion 3: Child needs or gets special therapy, such as physical, occupational, or speech therapy** |
| One or more visits to another person trained to provide treatment or counsel, past 12 months |
| **Criterion 4: Child has emotional, developmental, or behavioural problem for which he/she needs treatment or counselling** |
| One or more visits to a psychiatrist/psychologist, past 12 months |
| **Criterion 5: Child is limited or prevented in any way in his/her ability to do the things most children of the same age can do** |
| *Criterion omitted* |
